# A Systematic Review of Reporting Practices in Psychedelic Clinical Trials: Psychological Support, Therapy, and Psychosocial Interventions

**DOI:** 10.1089/psymed.2023.0007

**Published:** 2023-12-13

**Authors:** William Brennan, Alex R. Kelman, Alexander B. Belser

**Affiliations:** ^1^ Consultants, New York, NY, USA.; ^2^ Cybin, Inc., Toronto, Canada.; ^3^ UCLA Department of Psychiatry and Biobehavioral Sciences, Los Angeles, CA, USA.; ^4^ UCLA Department of Psychology, Los Angeles, CA, USA.

**Keywords:** psychedelic-assisted therapy (PAT), psychosocial interventions, clinical trials, reporting practices, treatment outcomes

## Abstract

**Background:**

Psychedelic-assisted therapy has gained significant attention in recent years. However, there is a lack of empirical clarity on the role of psychosocial interventions (PIs) in clinical trials of psychedelic treatment due in part to deficiencies in reporting practices found in the existing literature. These PI include non-drug support or interventions provided by psychotherapists or facilitators during all phases of treatment, sometimes called “psychological support,” “monitoring,” “psychedelic-assisted therapy,” or “psychedelic-assisted psychotherapy.” A brief review of recent research, historical studies, safety considerations, and participant perspectives suggests that PI has a substantive and critical impact on treatment outcomes.

**Methods:**

This systematic review examines the reporting practices on PI in published clinical trial results. The review employs a search of PubMed/Medline and PSYCinfo databases to identify relevant articles. It includes quantitative clinical studies treating patients with psychiatric indications using classic psychedelics (psilocybin, LSD, DMT, ayahuasca) or empathogenic drugs (MDMA) since 2000. The analytic approach follows a modified version of assessment items based on CONSORT extension statement and TIDieR checklist.

**Results:**

Thirty-three published psychedelic clinical trials met criteria. The review reveals that many published reports on psychedelic clinical trials did not report basic aspects of the intervention: 33% did not report the number of sessions, 45% did not report the duration of sessions, 42% did not report provider credentials, 52% did not report whether their intervention used a therapy manual, 64% did not reference a manual that was available to readers, and 82% did not report that they assessed treatment fidelity. A comparison with non-psychedelic trials shows that psychedelic trial reports underreport on key items related to PI.

**Discussion:**

The study highlights the problems of underreporting and the importance of improving reporting practices regarding PI in psychedelic clinical trials to enhance research standardization and improve treatment outcomes. Recommendations for improving reporting practices are provided.

## Introduction

As psychedelic-assisted therapy (PAT) treatments continue toward regulatory consideration and possible approval, the non-drug psychosocial interventions (PIs; defined here to include “psychological support,” “monitoring,” “psychedelic-assisted therapy,” or “psychedelic-assisted psychotherapy”) of these treatments remain understudied and poorly characterized.^1–5^

In these combination treatments, which include both study drug and supportive psychological intervention, PI include the presence of psychotherapists or facilitators involved in treatment sessions, the specific interventions they employ, and any supportive psychoeducational materials or digital technologies used in the treatment. Scant literature exists that facilitates an appraisal of how the presence or absence of these PI predicts treatment efficacy and safety. The unavailability of such data constitutes a barrier to discerning the specific components of these treatments that contribute to their efficacy and undermines their status as standardized treatments.^3,6,7^

One possible reason for this gap in our collective understanding is a general deficiency in reporting practices for the PI used in PAT clinical trials to date. It is common for published materials to omit information about the PI provided to study participants, such as basic information about their content (e.g., the specifics of the therapeutic approach used)^8^ and their quantity (e.g., number and length of non-drug preparation and integration sessions, which provide additional treatment support before and after dosing sessions). Without this information, a rigorous appraisal of PAT trial results is not possible, and any attempt to replicate the outcomes of these trials in follow-up studies or real-world treatment settings is needlessly hampered.

This article provides the first systematic review of the quality of reporting regarding PIs associated with PAT clinical trials. It is intended to identify areas for improvement so that reporting practices in the field can foster greater transparency, standardization, and rigor, ultimately enhancing the credibility and generalizability of psychedelic research and facilitating its integration into accepted therapeutic practices.

## Rationale for Increased Consideration of PIs

Before presenting the results of our analysis, we review findings in this section that support the contention, long held by many PAT experts,^9–26^ that PI are impactful factors in PAT treatment worthy of appropriate attention in discussions of trial outcomes.

### Therapeutic alliance predicts greater reductions in depression

Murphy et al.^27^ assessed the impact of therapeutic alliance on participant outcomes in a clinical trial investigating the efficacy of a PAT treatment involving two psilocybin administrations for moderate to severe depression. They found that therapeutic alliance going into the first psilocybin session predicted greater pre-session rapport, which contributed to higher scores on a measure of emotional breakthrough during this session and, in turn, greater reductions in depressive symptoms at 6 weeks.

Greater emotional breakthrough during the first psilocybin session also predicted increased therapeutic alliance leading into the second session. This stronger alliance led to a reduction in depressive symptoms at 6 weeks post-treatment both on its own and by way of its contributions to pre-session rapport and higher scores on a measure of mystical experience.

This study provides the first direct evidence for the therapeutic alliance's ability to directly contribute to clinical outcomes in PAT, similar to what has been observed across various traditional psychotherapy approaches,^28^ including combination therapies for depression.^29^

It also shows how several key psychotherapeutic elements—therapeutic alliance, rapport, emotional breakthrough—can interact in a mutually reinforcing manner that mirrors a process observed in traditional psychotherapy: a participant who feels a stronger working relationship with their therapist feels more comfortable exploring challenging personal material in the therapist's presence, which further develops the strength of the working alliance and enhances the efficacy of the treatment.

### High-support conditions versus low support conditions promote positive outcomes

Although no PAT clinical trial in this current wave has conducted a head-to-head comparison between a psychotherapy and a no-psychotherapy condition, one study has come close. Griffiths et al.^30^ designed a trial to determine, in part, any added benefits to providing a high-support (35 h) as compared with a low-support condition (only 7 h and 20 min). The trial consisted of two psilocybin sessions with healthy volunteers.

Participants were randomized into three conditions: (1) a “very low-dose” with “standard support” arm: 1 mg/70 kg psilocybin sessions with a total of 7 h and 20 min of non-drug “spiritual support” before and after the psilocybin sessions; (2) a “high-dose” with “standard-support” arm: 20–30 mg/70 kg psilocybin sessions with the same amount of spiritual support; and (3) a “high-dose with “high-support” arm: 20–30 mg/70 kg psilocybin sessions with an augmented total of 35 h of spiritual support.

Spiritual support consisted of staff-provided reading materials on meditative practice, a self-reflection journal, and sessions with study facilitators that consisted of instructions for preparing for psilocybin administrations and, subsequently, support in developing and maintaining a personal spiritual practice. The standard-support conditions (groups 1 and 2) consisted of six in-person meetings and two brief teleconferences with study facilitators over the course of the 2-month treatment. The high-support condition (group 3) included many more sessions: 18 in-person meetings with study facilitators and 8 group sessions over the course of 6 months. Notably, this support was not psychotherapy.

At the 6-month follow-up, the high-support group participants scored significantly higher than both standard-support groups on measures of altruistic/positive social effects, positive behavior changes, spirituality, engagement in journaling and other reflective practices, and enduring personal significance of their psilocybin experiences.

While these results in and of themselves do not represent a reduction in psychiatric symptoms, they reflect improvements on measures of well-being that have relevance to psychiatric functioning. Thus, while “spiritual support” is not psychotherapy *per se*, these results support the notion that the amount of non-drug support provided as an adjunct to psilocybin administration can influence the observed efficacy of PAT.

### Other relational elements found to influence outcomes

A survey study by Kettner et al.^31^ assessed psychological well-being and social connectedness in 886 attendees of psychedelic retreats before, during, and after drug administration. Using correlational and multiple regression (path) analyses, they found that several relational components of participant experiences—pre-ceremony rapport, perceived emotional support from facilitators and other attendees, and instances of self-disclosure—predicted feelings of “perceived togetherness and shared humanity”^31(p. 1)^ during ceremonies and retreats, which the authors dubbed experiences of “communitas.” In turn, these experiences predicted greater psychological well-being and social connectedness at 4 weeks post-retreat. So, while this psychedelic usage occurred outside of a clinical setting, three relational elements of psychotherapeutic process were found to predict clinically relevant outcomes.

Another survey study by Haijen et al.^32^ used a similar methodology to identify predictors of increased well-being after an instance of psychedelic use. They found that “setting,” a factor that consisted of two items pertaining to the perceived quality of the user's relationship to others present and attending to them during the experience and one item assessing perceptions of the environment, predicted higher well-being scores 2 weeks after the psychedelic experience. In this non-clinical setting, a supportive relational container predicted participant well-being.

### Historical comparison of 1960s psychedelic studies with and without psychotherapy

In his seminal work on the reasons behind the cessation of the first wave of PAT research, Oram^33^ provided another source of support for the importance of PI. He notes that the passage of the 1962 Kefauver-Harris Amendment to the Federal Food, Drug, and Cosmetic Act introduced a stricter research paradigm to PAT researchers. The researchers who continued to conduct trials tended to design clinical trials in which psychedelics could be studied within a paradigm that included little to no psychotherapy.

Oram reviewed the only four clinical trials investigating the use of LSD to treat alcoholism to be funded by the National Institute of Mental Health between 1963 and 1968. The first two trials^34,35^ used no psychotherapy beyond occasional offerings of reassurance. Hollister et al. found that “LSD produced slightly better results”^35(p. 1352)^ than placebo at 2 months, but the effect quickly faded thereafter. Ditman et al. also found no durable results, noting that “LSD did not uniquely produce the traditional ‘therapeutic’ experience but appeared to be surpassed in that area by methylphenidate.”^34(p. 1)^

The third trial^36^ compared four conditions. One group got no drug and no psychotherapy, a second received LSD with no psychotherapy, and the other two groups received LSD and one of two psychotherapeutic interventions (based on either psychoanalysis or hypnotherapy) in which participants were engaged for only the first 2 h of the LSD session before being left alone for the remaining duration of the drug effects. No significant differences were found between the four groups. In summary, the first three studies with little or no therapy provided null results.

The fourth trial^37^ included robust PIs based on previous PAT approaches. Participants received 20 h of preparatory psychotherapy before drug administration sessions. Also, the support that participants received during drug sessions lasted the full duration and was more thoughtfully tailored to the needs of individuals in altered states of consciousness.

Results demonstrated therapeutic efficacy: participants in the experimental group showed significant improvements in problem drinking behavior at 6 months compared with the control group. Oram^33^ presented these four studies in tandem to argue that PIs can exert a pivotal impact on treatment efficacy across trials that assess the same drug for the same clinical indication.

### Participant voices

Another source of evidence for the importance of PI in PAT trials comes from the experience of participants in PAT clinical trials and psychedelic-assisted treatment in comparable contexts, such as facilitated retreats. Several qualitative studies that have inquired about the role of the facilitator-participant relationship have found unanimous or near-unanimous support for its importance.^38^

Lafrance et al. found that “all of the [16] participants we interviewed […] emphasized the importance of what they perceived as safe ceremonial structures and leadership to maximize potential healing and to minimize risks or harms.”^39(p.5)^ Similarly, Watts et al. found that, “Overall, support from skilled guides was seen as a key part of the intervention [by 17 out of 20 patients].”^40(p.550)^ Noorani et al. also reported that:

All [twelve] participants recognised the importance of trusting and developing rapport with their session guides as necessary preparation for navigating experiences in psilocybin sessions. Moreover, ten participants identified rapport with the study team during the preparatory counselling as a crucial factor in [successful outcomes].^41(p.761)^

The specific participant quotes presented in these studies emphasize themes of safety and interpersonal support. One participant stated, “I think if you didn't have [the rapport with facilitators], I'm not sure if it would work—when you know that people want you to do well…want this to work for you.”^41(p.762)^ Another spoke to the importance of the safety provided by session facilitators, noting:

You know safety is 100% important because you cannot resolve trauma if you do not feel safe, because safety is part of the trauma. So really feeling like the people holding space are doing it in a very adept way.^39(p.5)^

### Safety concerns: adverse events

The potential importance of PIs in establishing participant safety was also discussed by the authors of a recent review of adverse events (AEs) in PAT clinical trials.^42^ They stated that “treatment designs that reduce or minimize positive contextual components (e.g., time spent preparing patients, number of therapists, strength of the therapeutic relationship, time spent providing aftercare and integration) may increase the incidence of AEs,”^42(p. 14)^ citing agreement with earlier expert opinions.^33,43,44^

Elsewhere, authors of a case report on a patient who experienced a severe adverse reaction to non-medical psilocybin use similarly argued that “regulators may need to consider a requirement that certain minimum safety and psychotherapy protocols be implemented”^45(p. 894)^ in settings that provide PAT to protect against the potential harms resulting from idiosyncratic drug reactions. The perspective found in these two publications resonates with those of other experts^21,46^ who have argued that safe provision of PAT rests upon sufficient and appropriate PI.

However, it would be incautious to posit a formulaic relationship between safety and PI that equates “more” with “safer.” One trial participant's account suggests that an excess of poorly boundaried support from her therapists cultivated a harmful dependency that may have contributed to the dynamics that led to sexual misconduct.^3,47,48^

Several other participants have reported similar feelings of strong dependency on PAT trial therapists, which they attributed to the combined effects of the MDMA and the intensive support provided. These participants claimed that this dependency contributed to severe post-trial deteriorations in psychiatric functioning.^48^ Their perspectives caution us against any simplistic reading of the relationship between PI and safety in PAT, while lending weight to the contention that this relationship demands more thorough and nuanced consideration.

## Systematic Review of Current Reporting Practices

### Methods

#### Inclusion criteria

We aimed at reviewing all publications associated with quantitative clinical studies that involved the treatment of patients with a psychiatric indication with either a classic psychedelic (e.g., psilocybin, LSD, DMT, ayahuasca, etc.) or an empathogenic drug (MDMA) published in English since 2000, including open-label studies and randomized controlled trials, to assess their PI reporting practices.

We excluded reviews, survey-based studies, secondary analyses, and studies with healthy volunteers. We selected these criteria to include only those publications that served as the primary publication reporting on the clinical efficacy of a PAT intervention.

Regarding ketamine, its enantiomers, and related compounds: as their use in clinical trials typically lacks emphasis on concurrent PIs, and as the neurobiological mechanisms of ketamine are fundamentally distinct from that of classic psychedelics and empathogens, we excluded these compounds from the scope of this review.

#### Data collection

We systematically searched the PubMed/Medline and PSYCinfo databases on May 26, 2023. We used two simultaneous search strings, one containing the names of psychedelic compounds and the other containing study type (i.e., “clinical trial”). Due to the similarity in our inclusion criteria to that of Breeksema et al.,^42^ we modified the search terms used in their study. We excluded from their terms any that pertained to qualitative studies and case reports, as our study differed in its exclusive focus on clinical trial reports. See Supplementary Data for a detailed list of search terms. The time range of the search was from 2000 to May 2023. Our systematic search was corroborated by checking reference lists of reviews with similar inclusion criteria.^42,49^

#### Outcomes of interest

Following Candy et al.,^50^ who conducted an analysis of reporting in randomized controlled trials of multicomponent interventions in non-psychedelic clinical trials, our intention was to evaluate the extent to which the selected articles' descriptions of their interventions facilitate (1) replicability of the intervention in clinical practice or in follow-up trials, and (2) an assessment of the generalizability of trial outcomes.

To assess these areas of interest, Candy et al.^50^ developed a list of 19 assessment items based on the publication standards set forth in the CONSORT extension statement^51^ and the TIDieR checklist,^52^ two widely used sets of guidelines for preparing reports of trial results that are endorsed by many high-impact biomedical journals.^53,54^

Our analysis used a modified version of this list of assessment items. We evaluated 16 of the original items in a way that mirrored the approach of the original authors. The item “comparator arm” was excluded from our analysis, since all PAT trials meeting our criteria to date have attempted to keep the PI consistent across drug and placebo groups, rendering this item moot. The “how” item, which the original authors used to assess whether or not a report described the procedures used in an intervention, was determined to be a redundant item that referred globally to information that was assessed more finely by other items (e.g., supplemental materials).

The “word count” item was initially assessed, but we found that the results failed to reflect the variety of ways in which authors provided details about PI without adding text to primary and supplementary materials (e.g., referencing a publicly available manual). We also added two new items that captured additional important aspects of PI reporting in psychedelic clinical trials: “therapy manual available” and “descriptor for non-drug sessions.” See Table 1 for item definitions.

**Table 1.** Description of Items Extracted

Item label	Description of item reported in the publications
Brief name	Multicomponent intervention is given a simple name.
Rationale	Rationale, theory, or goal for testing the intervention.
Treatment setting	Information about where study sessions occurred (e.g., hospital outpatient unit).
Recipient characteristics	Description of participant inclusion and/or exclusion criteria.
Provider credentials	Information on the credentials of the provider administering the treatment.
No. of sessions	Sufficient information to determine how many non-drug sessions occurred. If this varied across participants, an explanation of how decisions to vary were made and an assessment of any impact on outcomes.
Duration of sessions	Sufficient information to determine how long non-drug sessions lasted. If this varied, an explanation of the rationale for this variability and an assessment of its impact on outcomes.
Timeline of sessions	Sufficient information to determine how much time elapsed between sessions.
Therapy manual referenced	Acknowledgment that a standardized therapist/facilitator manual specific to this intervention was used in the study.
Supplementary materials	Availability of additional materials that further describe non-drug elements (inclusive of publicly available therapy manuals).
Therapy manual available	Open availability of a manual that details the therapeutic approach used.
Intervention supporting materials	Provision or description of any therapist training materials or supportive psychoeducational materials for participants used in the intervention.
Descriptive aids	Figures or tables in publication that further explain the combined intervention.
Tailoring to participants	Indication that intervention was tailored to the needs or one or more specific participants without a global modification to the protocol.
Intervention modifications	Description of any protocol modifications made during the study.
Fidelity assessed	Indication that fidelity or adherence ratings were conducted for the combined intervention.
Fidelity outcome	Reporting on the outcome of the fidelity assessment.
Patient views	Acknowledgment of patient feedback incorporated in intervention development.
Descriptor for non-drug sessions	Term used in published materials to describe support provided to participants by facilitators in non-drug sessions.

The first 18 items are included in Table 2. The final item is included in Table 3.

Numbers in parentheses are percentages. Shaded cells indicate a value at least 10 percentage points lower than that of comparator (psychedelic trials vs. combined non-psychedelic trials). “—” indicates no data are available for this item.

^a^ Seven trials referenced versions of the same publicly available manual,^55,56^ whereas another^57^ referenced a four-page section of the study appendix meant to “coordinate the therapeutic procedure” of “active [study] personnel who are also familiar with the method of psycholytic (i.e., psychedelic) therapy”.

^b^ Fifteen of these descriptive aids were study timelines presenting the order of study visits.

^c^ These instances of tailoring include varying the study drug dose per participant preference, offering additional support sessions to accommodate post-dosing session distress, employing rescue medications for anxiety or nausea during dosing sessions, and inpatient hospitalizations after dosing sessions.

^d^ As a percentage of studies that assessed fidelity, reported in the previous row.

#### Data handling and analysis

One author extracted the data (W.B.), whereas the others (A.R.K., A.B.B.) reviewed these findings independently. Discrepancies were discussed and resolved. All but one item was coded on whether it was adequately reported, scoring “1” if yes or “0” if not. The remaining item (“descriptor for non-drug sessions”) was assessed qualitatively. Our search for the presence of these items included primary publications and any associated supplementary materials. The outcomes of this analysis are presented in Tables 2 and 3.

**Table 2.** Frequency of Psychotherapeutic Element Reporting in Published Clinical Trial Results (Total (%))

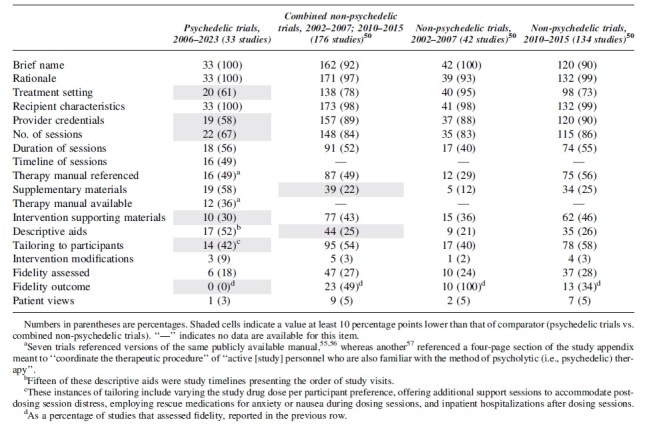

**Table 3.** Descriptions of Psychotherapeutic Interventions in Publications Associated with Psychedelic Clinical Trials

Pub. year	First author	Drug	Indication	Descriptor(s) for non-drug sessions
2006	Moreno^58^	Psilocybin	Obsessive-compulsive disorder	Unnamed support
2008	Bouso^59^	MDMA	Posttraumatic stress disorder	“Psychotherapeutic/psychotherapy sessions”
2011	Grob^60^	Psilocybin	Anxiety in context of advanced-stage cancer	Unnamed support
2011	Mithoefer^61^	MDMA	Posttraumatic stress disorder	“Psychotherapy sessions”
2013	Oehen^62^	MDMA	Posttraumatic stress disorder	“Psychotherapy sessions”
2014	Gasser^63^	LSD	Anxiety due to life-threatening illness	“Psychotherapy sessions”
2014	Johnson^64^	Psilocybin	Tobacco addiction	“Preparation, integration, and support meetings,” “Cognitive behavioral therapy”
2015	Bogenschutz^65^	Psilocybin	Alcohol use disorder	“Psychosocial intervention,” “Motivational enhancement therapy”
2016	Carhart-Harris^66^	Psilocybin	Treatment-resistant depression	“Psychological support”
2016	Griffiths^67^	Psilocybin	Psychiatric diagnoses w/anxiety or mood symptoms in context of life-threatening cancer	“Meetings”
2016	Ross^68^	Psilocybin	Anxiety disorders in context of life-threatening cancer	“Targeted psychotherapy”
2016	Sanches^69^	Ayahuasca	Major depressive disorder	Unnamed support; 2-week inpatient hospitalization
2018	Danforth^70^	MDMA	Social anxiety in the context of autism	“Preparatory and integrative psychotherapy,” “Mindfulness-based therapy adapted from dialectical behavioral therapy”
2018	Mithoefer^71^	MDMA	Posttraumatic stress disorder	“Psychotherapy sessions”
2018	Ot'alora^72^	MDMA	Posttraumatic stress disorder	“Psychotherapy sessions”
2019	Palhano-Fontes^73^	Ayahuasca	Treatment-resistant depression	Unnamed support; 1-week inpatient stay as needed
2020	Anderson^74^	Psilocybin	AIDS-related demoralization	“Group therapy” using “modified supportive expressive group therapy”
2020	Monson^75^	MDMA	Posttraumatic stress disorder	“Cognitive behavioral conjoint therapy sessions”
2020	Wolfson^76^	MDMA	Anxiety due to life-threatening illness	“Psychotherapy sessions”
2021	Carhart-Harris^77^	Psilocybin	Major depressive disorder	“Psychological support” in primary report; “Accept connect embody model” in supplemental materials
2021	Davis^78^	Psilocybin	Major depressive disorder	“Supportive psychotherapy”
2021	Dos Santos^79^	Ayahuasca	Social anxiety disorder	Unnamed support
2020	Jardim^80^	MDMA	Posttraumatic stress disorder	“Psychotherapy sessions”
2021	Mitchell^81^	MDMA	Posttraumatic stress disorder	“Therapy sessions”
2021	Sessa^82^	MDMA	Alcohol use disorder	“Psychological support,” “Psychotherapy sessions”
2022	Bogenschutz^83^	Psilocybin	Alcohol use disorder	“Psychotherapy sessions,” “Motivational interviewing and cognitive behavioral therapy for AUD”
2022	D'Souza^84^	DMT	Major depressive disorder	“Strategic psychoeducation/support”
2022	Goodwin^85^	Psilocybin	Treatment-resistant depression	“Psychological support”; digital adjuncts mentioned in suppl. materials
2023	Holze^57^	LSD	Anxiety disorders, or significant anxiety in context of life-threatening illness	“Talking psychotherapy”
2023	Shnayder^86^	Psilocybin	Major depressive disorder in context of cancer	“Therapeutic sessions”
2023	Schneier^87^	Psilocybin	SSRI-resistant body dysmorphic disorder	“Psychological support”
2023	Sloshower^88^	Psilocybin	Major depressive disorder	“Psychotherapy sessions,” “Psychotherapeutic support”
2023	von Rotz^89^	Psilocybin	Major depressive disorder	“Psychological support”

SSRI, Selective serotonin reuptake inhibitor.

Data from Candy et al.^50^ were added to Table 2 to facilitate a comparison with reporting on non-psychedelic multicomponent interventions whenever comparable data were available for an item. However, there are several caveats to note regarding this comparison. First, the original authors assessed reporting on interventions designed to promote therapy adherence rather than combination pharmacotherapies.

Second, while our analysis searched for the presence of the items in primary and supplementary materials, Candy et al.^50^ searched only within the primary publications. Finally, the publication dates of the trials we assessed ranged from 2006 to 2023, whereas the original authors looked at studies published within two date ranges: 2002–2007 and 2010–2015.

### Results

**Fig. 1.**
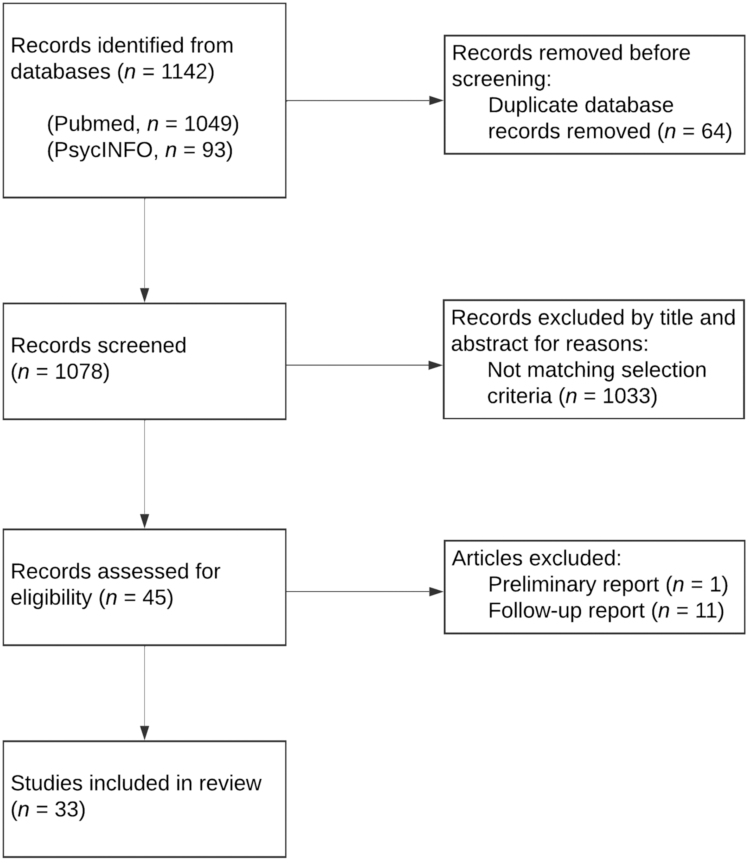
Data selection process.

We found 1142 articles (PubMed, *n* = 1049; PsycINFO, *n* = 93). After removal of duplicates, the remaining 1078 titles and abstracts were screened independently by the first author. After removing those that did not meet inclusion criteria, the full text of 45 articles were assessed for eligibility, and an additional 12 articles were excluded: secondary analyses of data from other included publications (11) and preliminary reports on results that were later presented in full in other included publications (1). After assessment, 33 articles were included (see Fig. 1 for process).

The final list of included studies (Table 3) was agreed upon by the second and third authors without any discrepancies. Risk of bias in these studies was not assessed, as it was not pertinent to our purpose of analyzing the quality of reporting practices.

The results in Table 2 reflect the extent to which the assessed items were included in reports on PAT clinical trials. When comparing psychedelic clinical trial reporting to the reference of non-psychedelic clinical trial reporting: for 6 out of 16 of the items for which data from non-psychedelic clinical trials are available, PAT trial reports lagged behind this comparator by at least 10 percentage points.

These items include “treatment setting,” “provider credentials,” “number of sessions,” “intervention supporting materials,” “tailoring to participants,” and “fidelity outcome.” The latter three items are particularly significant to the reporting of non-drug PI. On only two items (“descriptive aids” and “Supplementary Materials”) did psychedelic trial reports outperform non-psychedelic trials by at least 10 percentage points.

Commonly found issues that contributed to lower aggregate scores for psychedelic trials on Table 2 include:

- Lack of reporting regarding number of non-drug sessions, despite across-the-board reporting on the number of dosing sessions
- Lack of reporting about the timeline of preparatory and integration sessions
- Lack of reporting about the duration of integration sessions, even when duration of other sessions was provided
- Indications that additional sessions or other relevant supports were provided for some or all participants beyond the clearly defined session schedule in the absence of reporting on the frequency or criteria used to determine their provision
- Discrepancies between information in primary publications and their associated supplementary materials, such as differing number of sessions, duration of sessions, or non-drug intervention nomenclature (e.g., “debrief” vs. “integration”)
- References to non-psychedelic manualized therapies that influenced the therapeutic approach of the study with little information on how they were adapted for use within a PAT trial
- Reliance on the standardization of incorporated non-psychedelic therapeutic approaches rather than standardizing the adapted approach used in a specific PAT trial

### Discussion

In this study, we aimed at systematically reviewing the reporting of PIs in published clinical trials of psychedelic treatment. We identified significant deficiencies. Of the 33 psychedelic clinical trials, 42% did not report provider credentials, 52% did not report whether their intervention used a therapy manual, 64% did not reference a manual that was available to readers, 33% did not report the number of sessions, 45% did not report the duration of sessions, 82% did not report that they assessed treatment fidelity, and 100% did not report treatment fidelity outcomes.

On most of these items, reports of psychedelic trials underperformed those of non-psychedelic trials^50^ by margins of up to 31%. These omissions pose challenges to research reproducibility, assessment of treatment efficacy, and replication of outcomes in follow-up research and treatment settings.

The widespread underreporting of PI in current publications hampers our understanding of the factors contributing to treatment outcomes in PAT. It is crucial to recognize the impact of PI on treatment factors that may influence outcomes, such as therapeutic alliance and participant feelings of safety, as suggested by previous research and participant perspectives. Neglecting to report these elements can undermine the validity and generalizability of study findings, hindering our ability to evaluate the efficacy and safety of PAT. Ethical research also requires adequate reporting of trial methodology that supports our collective ability to confront the replication crisis that has beset the psychological sciences for decades.^90^

While the amount of PI provided varies from trial to trial, thus far, even minimal psychological support constitutes a substantial PI that merits a full description. The field must recognize the significance of PI and strive for research standardization to promote better understanding, replication, and implementation of PAT.

The reasons for this deficiency remain speculative. The deficiencies in reporting practices observed in this study may be attributed, in part, to publication conventions and the emphasis on pharmacotherapies in the journals in which psychedelic trials are often published.^8^ This contention likely accounts for some of the inadequacy in PI reporting, as very few PAT trial reports are published in psychotherapy journals that are more likely to have word limits that allow for rich descriptions of PI.

As such, the focus in reporting has largely been on the pharmacotherapy rather than non-drug elements of treatment. Although PAT is a combination intervention, most trials are designed to vary drug administration conditions while holding the psychosocial aspects constant. This and a number of other incentives may lead authors to publish in journals more disposed toward pharmacotherapies than psychotherapies.

Some authors have worked around this limitation by providing more details about PI in their supplemental materials. Accordingly, PAT trials surpassed non-psychedelic trials on the “supplementary materials” item on Table 2 by a significant margin. To the extent that authors' inadequate reporting on PI is due to their choice of journal, this strategy offers an effective workaround.

The reporting deficiencies also occur in the context of strong incentives to reduce the expected cost of PAT treatment in preparation for commercialization^91^ by downplaying the role of PIs in favor of focusing on the pharmacological intervention alone. With psychedelic clinical trials, the absence of adequate reporting leads to a lack of data to fully represent the nature of the intervention. Without these data, any future trial designs that omit psychosocial support in psychedelic trials (e.g., a 2 × 2 factorial design or dismantling trial that eliminates the therapy in one of the arms) may adversely affect outcomes, potentially posing safety risks and jeopardizing clinical efficacy.

While the contribution of the non-drug therapeutic elements of treatment has not been formally studied, possibly due to a lack of regulatory and funding priorities, a variety of evidence suggests that providing robust psychosocial support improves outcomes. Murphy et al.^27^ found that therapeutic alliance, a key component of PI, predicts improved outcomes in a trial involving psilocybin treatment for depression.

Further evidence comes from a trial by Griffiths et al.,^30^ which compared a high-support (35 h) with a low-support condition (only 7 h and 20 min). At 6 months post-treatment, the high-support group participants scored significantly higher on measures of well-being, implying that the amount of non-drug support can influence PAT efficacy.

Additional studies have identified other relational elements, such as pre-ceremony rapport and perceived emotional support, as predictors of improved outcomes.^31,32^ Finally, a historical analysis of 1960s psychedelic studies compared four clinical trials and concluded that only the trial containing robust psychotherapeutic intervention showed significant improvements in treatment outcomes.^33^ Many qualitative studies have also reported the importance that participants place on the facilitator-participant relationship, emphasizing themes of safety and interpersonal support.^38–41^

In service of improved PI reporting, we offer a list of recommendations to improve reporting practices in psychedelic clinical trials based on the current review's assessment using items from the TIDieR checklist The list is not as comprehensive as the CONSORT guidelines, but is intended to augment and clarify reporting for psychedelic clinical trials, as it is based on observations about specific deficits in the PAT literature discovered through the process of conducting this review. These recommendations reflect the elements that authors have most often missed to date. They are provided in Table 4.

**Table 4.** Recommendations to Improve Reporting Practices in Psychedelic Clinical Trials Based on the Current Review's Assessment Using Items from the TIDieR Checklist

Deficient item	Recommendation for improved reporting practices
1. Treatment setting	Provide details on the treatment setting, including a rich description of the space used for dosing sessions.
2. Provider credentials	Describe provider credentials and any other trial-relevant training they underwent.
3. Number of sessions	Report the average number of non-drug sessions provided and note any impact of variability on treatment outcomes.
4. Duration of sessions	Report the average number of total minutes of non-drug support provided by therapists/facilitators (including screening visits, intakes, follow-up calls, informed consent review, and/or other clinician-administered or self-report assessments conducted with facilitators).
5. Timeline of sessions	Provide more details on when non-drug sessions occurred, perhaps using a timeline with numbered days.
6 Therapy manual referenced/available	Note how non-drug psychosocial interventions were standardized and, when feasible, provide access to trial-specific manuals or other materials used in this process.
7. Intervention supporting materials	Describe or provide any psychoeducational materials or technology platforms used with participants (e.g., preparatory hand-outs or videos).
8. Tailoring to participants	Indicate and provide rationale for any variability in non-drug support and/or provider contact.
9. Fidelity assessed/outcome	Assess fidelity to the complete multicomponent intervention and report outcomes.

#### Limitations

While this study provides insights into the reporting practices of PIs in psychedelic clinical trials, it is important to acknowledge several limitations. First, the search was conducted in English-language databases, which may introduce language bias and exclude relevant studies published in other languages.

Second, the search terms and databases selected may have overlooked some relevant publications with psychedelic analogues, potentially affecting the comprehensiveness of the review. Third, data extraction was performed by a single author, which may introduce subjectivity and potential errors. However, the review process involved independent reviews by two authors to minimize bias and enhance reliability. The authors arrived at final results through a process of discussion and consensus.

Fourth, though the results reflect the frequency with which authors referred to supplemental materials or publicly available therapy manuals as sources of additional information about PI, they do not capture the use of separate, secondary publications to convey this data.^46,92,93^ Also, our results did not capture authors' references to other publications that discuss PI in PAT more generally.^14,21^ We determined that our quantitative approach could not capture the diversity of ways in which these outside publications were used as adjunctive means of describing PI. These outside references varied significantly in terms of their utility in accurately describing the PI of the specific study that referenced them and their timeliness in relation to the publication of the primary report, with many being published years after the results of the trial. However, in some instances, these references could be considered helpful ways of describing PI that were not represented in the data in Table 2.

## Conclusion

In conclusion, this systematic review of reporting practices on PIs in psychedelic clinical trials reveals significant deficiencies in the reporting of these crucial components. The study demonstrates that many published reports on psychedelic clinical trials lack essential details regarding the quantity and quality of PI, including the number and duration of sessions, provider credentials, use of therapy manuals, and assessment of treatment fidelity. A comparison with non-psychedelic clinical trial reporting highlights the underperformance of psychedelic trial reports on key reporting items related to PI.

The inadequate reporting of PI in current publications poses challenges to research reproducibility, the assessment of treatment efficacy, and the implementation of PAT in real-world settings. To address the reporting gap, psychedelic clinical trial reports should minimally include the nine suggested elements (Table 4) in future publications. In conclusion, the findings of this review underscore the importance of full and comprehensive reporting on PI in psychedelic clinical trials to enhance research standardization and improve treatment outcomes.
