# Supplementary material for: A Systematic Review of Reporting Practices in Psychedelic Clinical Trials: Psychological Support, Therapy, and Psychosocial Interventions
Source: Psychedelic Med (New Rochelle). 2023 Dec 13;1(4):218–29. doi: 10.1089/psymed.2023.0007 (PMC11658666; doi:10.1089/psymed.2023.0007)
Supplement: Supplementary Data [file psymed.2023.0007_suppl_data.docx]

**PSYCINFO**

(DE "Hallucinogenic Drugs" OR DE "Bufotenine" OR DE "Lysergic Acid Diethylamide" OR DE "Mescaline" OR DE "Peyote" OR DE "Phencyclidine" OR DE "Psilocybin" DE "Lysergic Acid Diethylamide" OR DE "Mescaline" OR DE "Methylenedioxymethamphetamine" OR TI (“hallucinogen*” OR “bufotenine” OR “mescaline” OR “peyote” OR “phencyclidine” OR “psilocybin” OR “lysergic acid diethylamide” OR N-methyl-3,4-methylenedioxyamphetamine” OR “banisteriopsis” OR “N,N-dimethyltryptamine” OR “mescaline” OR “LSD” OR “ayahuasca” OR “methylenedioxymethamphetamine” OR “MDMA” OR “dimethyltryptamine” OR “DMT OR “5-MeO-DMT” OR “psychedelic” OR “hallucinogen*”) OR AB (“hallucinogen*” OR “bufotenine” OR “lysergic acid diethylamide” OR “mescaline” OR “peyote” OR “phencyclidine” OR “psilocybin” OR N-methyl-3,4-methylenedioxyamphetamine” OR “banisteriopsis” OR “N,N-dimethyltryptamine” OR “mescaline” OR “ayahuasca” OR “methylenedioxymethamphetamine” OR “MDMA” OR “dimethyltryptamine” OR “5-MeO-DMT”)

**AND**

(DE "Clinical Trials" OR DE "Randomized Controlled Trials" OR TI (“clinical trial*" OR “random*” OR “trial” OR “controlled stud*” OR “clinical-stud*” OR “random*” OR “trial” OR “clinical-stud*” OR “uncontrolled stud*” OR “open-label”)

**NOT**

(PO Animal NOT PO Human)

**NOT**

(Filter for time range 2000-2023)

**PUBMED**

("Hallucinogens"[Mesh] OR "Lysergic Acid Diethylamide"[Mesh] OR "Psilocybin"[Mesh] OR "N-Methyl-3,4-methylenedioxyamphetamine"[Mesh] OR "Banisteriopsis"[Mesh] OR "N,N-Dimethyltryptamine"[Mesh] OR "Mescaline"[Mesh] OR “Lysergic-Acid-Diethylamide”[tiab] OR LSD[ti] OR Psilocybin[tiab] OR Ayahuasca[tiab] OR MDMA[tiab] OR methylenedioxyamphetamine[tiab] OR dimethyltryptamine[tiab] OR DMT[ti] OR “5-MeO-DMT”[tiab] OR psychedelic[ti] OR hallucinogen*[ti])

**AND**

("Clinical Trial" [Publication Type] OR “controlled-stud*”[tiab] OR “open-label” [tiab] OR “uncontrolled-stud*”[tiab] OR “clinical-stud*”[tiab] OR random*[tiab] OR trial[tiab])

**NOT**

("Animals"[Mesh] NOT "Humans"[Mesh])

**AND**

(2000/1/1:3000/12/12[pdat])
